# Analysis of candidate genes expected to be essential for melanoma surviving

**DOI:** 10.1186/s12935-020-01584-2

**Published:** 2020-10-07

**Authors:** Irina A. Krivosheeva, Alexandra Yu. Filatova, Sergei A. Moshkovskii, Ancha V. Baranova, Mikhail Yu. Skoblov

**Affiliations:** 1 Laboratory of Functional Genomics, Research Centre of Medical Genetics, Erevanskaya Street, 10 building 2, Floor 44, Moscow, 115304 Russia; 2 Laboratory of Medical Proteomics, Institute of Biomedical Chemistry, Moscow, Russia; 3 School of Systems Biology, George Mason University, Fairfax, VA USA

**Keywords:** Melanoma, siRNA knockdown, Viability, Wound-healing assay

## Abstract

**Introduction:**

Cancers may be treated by selective targeting of the genes vital for their survival. A number of attempts have led to discovery of several genes essential for surviving of tumor cells of different types. In this work, we tried to analyze genes that were previously predicted to be essential for melanoma surviving. Here we present the results of transient siRNA-mediated knockdown of the four of such genes, namely, UNC45A, STK11IP, RHPN2 and ZNFX1, in melanoma cell line A375, then assayed the cells for their viability, proliferation and ability to migrate in vitro. In our study, the knockdown of the genes predicted as essential for melanoma survival does not lead to statistically significant changes in cell viability. On the other hand, for each of the studied genes, mobility assays showed that the knockdown of each of the target genes accelerates the speed of cells migrating. Possible explanation for such counterintuitive results may include insufficiency of the predicting computational models or the necessity of a multiplex knockdown of the genes.

**Aims:**

To examine the hypothesis of essentiality of hypomutated genes for melanoma surviving we have performed knockdown of several genes in melanoma cell line and analyzed cell viability and their ability to migrate.

**Methods:**

Knockdown was performed by siRNAs transfected by Metafectene PRO. The levels of mRNAs before and after knockdown were evaluated by RT-qPCR analysis. Cell viability and proliferation were assessed by MTT assay. Cell migration was assessed by wound healing assay.

**Results:**

The knockdown of the genes predicted as essential for melanoma survival does not lead to statistically significant changes in cell viability. On the other hand, for each of the studied genes, mobility assays showed that the knockdown of each of the target genes accelerates the speed of cells migrating.

**Conclusion:**

Our results do not confirm initial hypothesis that the genes predicted essential for melanoma survival as a matter of fact support the survival of melanoma cells.

## Introduction

Cancer is a group of abnormal cell growth diseases with the potential to invade or spread to other body locations. In USA, cancer is the second leading cause of death in both men and women of all ages [1]. Many treatments are directed against cancer: there are chemotherapy, immunotherapy, radiation, surgery, stem cell or bone marrow transplantation, hormone therapy and also a palliative care, and various combinations of two or more of these modalities [2]. A majority of these approaches are rid with severe side effects or are costly. Since patterns of spontaneous mutations are unique for each case, general approaches to cancer therapy affect healthy cells as well [3, 4]. One of the most promising approaches to the treatment of cancer is to treat each patient individually, or at least to treat each type of cancer in a specific way, in a framework of the personalized medicine [5]. A strategy when a common cytotoxic therapy is combined with target molecular therapy is highly promising too [6].

One of the most important premises of the personalized medicine is thorough understanding of underlining genetics. Genetics of cancer cells defines their resistance to drugs, aggressiveness and ability to metastasize. In particular, it is critical to understand the difference between cancer and normal cells genomes, distinguish them successfully and selectively destroy tumor cells.

A majority of the studies of cancer genetics concentrate on the genes either mutated [7] or differently expressed [8] in cancer cells when compared to adjacent normal tissues. Recent extended systematic review reported more than 200 potential growth-promoting oncogenes capable of driving 21 types of human tumors [9]. It is important to understand that the drivers of cancer proliferation are not identical to the genes essential for cancer surviving. In evolutionary process of tumor progression, cells with mutations in the genes essential for tumor survival will perish either due to permanent cell circle arrest or by elimination in the process of immune surveillance. On the contrary, intactness of the “essential” genes allows the cell to survive, divide and eventually end up in DNA or RNA sequencing lab. Targeting these genes in frame of the cancer therapy may yield so desired selective elimination. In an earlier study, an Abraham Wald's approach to identify vulnerabilities of aircrafts as the parts and the systems free of the bullet-made holes on landing was employed to highlight the genes and corresponding proteins never mutated in cancer cells and, therefore, essential for their survival [10]. Arguably, this Their approach better fits to the study of tumors driven by point mutations [11], such as lung tumors or melanomas, rather than by overall instability of chromosomal structure, common in the prostate cancer and the sarcomas of various types. In this light, initial study was performed on a model of cutaneous melanoma, one of the most aggressive and deadly skin cancers [12–14], with steadily rising cumulative incidence [15]. This type of cancer is well-studied, with many sequenced genomes available [10], thus, improving robustness of the predictions made in silico. Furthermore, melanoma has the highest somatic mutation frequency when compared to other cancers [16]. Based on groundbreaking approach which aggregates evolutional dN/dS parameter as a measure of negative selection with quantified gene expression and assessment of the functional impact of amino acid changes, our colleagues have predicted a set of 91 protein-coding genes potentially essential for survival of melanoma cancer cells.

Here, we assessed the role of some of these genes on survivability of melanoma cell line by performing their siRNA-mediated knockdown followed by investigation of the cell viability, proliferation and migration rate in vitro. While we hypothesized that melanoma cells lacking the transcripts of these essential genes would show decrease in both the proliferation and viability, experiments proved otherwise.

## Materials and methods

### Cell lines and cell culture conditions

Human malignant melanoma A375 and Human Embrionic Kidney HEK293T cells were cultured under standard conditions. In brief, Dulbecco's Modified Eagle's Medium (DMEM) with 10% fetal bovine serum (FBS) and l-glutamine were placed in humidified atmosphere with 5% CO2 at 37 °C. Before transfection, cells were trypsinized, resuspensed, diluted to 1 ml, and counted by Partec Flow Cytometer.

### Knocking down the expression of the genes in cell lines

siRNAs were designed according to recommendations [17] with the use of in-home software. The sequences of siRNAs are listed in Table 1. Knockdown efficiency of each of siRNAs was evaluated in HEK293T cells by RT-qPCR. The knockdown was performed as described in [18]. In brief, cells were grown up to 80% confluency, trypsinized, resuspended in antibiotics-free medium, seeded into 96-well plates at 10 x 103 cells/well and transfected with Metafectene (Biontex) according to manufacturer’s instructions: a 15 min incubated mixture of 0.4 mkl of Metafectene and 15 ng of siRNA in 60 mkl of PBS were added to cells in 96-well plates. Transfection efficiency was monitored by flow cytometry of cells transfected with FAM-tagged siRNA (siFlu) and exceeded 70% in all experiments.

**Table 1:** Sequences of primers and siRNAs used in the work

Primers of target genes	
RHPN2 (forward)	GGGCTGAACATCTCGGTGG
RHPN2 (reverse)	CCGGCTAGGCGTCCGACA
UNC45A (forward)	CTCCACTCTCAAACTGGCTAA
UNC45A (reverse)	GTCGGCATCAAAGGTCAGGT
ZNFx1 (forward)	TTGGAATTCTGCCAGCGAAC
ZNFx1 (reverse)	CCTGCGAGAAGATTTCGTCA
STK11IP (forward)	CTTGTTGGTGTGTCCCCTG
STK11IP (reverse)	GTGCGAGCTGCTTGGAGTT
HK genes primers and probes	
B2M (forward)	GAGTATGCCTGCCGTGTG
B2M (reverse)	AATCCAAATGCGGCATCT
B2M (probe)	FAM-CCTCCATGATGCTGCTTACATGTCTC-BHQ1
TFRC (forward)	GCCAACTGCTTTCATTTGTG
TFRC (reverse)	ACTCAGGCCCATTTCCTTTA
TFRC (probe)	ROX-AGGGATCTGAACCAATACAGAGCAGACA-BHQ1
HPRT (forward)	TCAGGCAGTATAATCCAAAGATGGT
HPRT (reverse)	AGTCTGGCTTATATCCAACACTTCG
HPRT (probe)	TAMRA-CAAGCTTGCTGGTGAAAAGGACCCC-BHQ1
TBP (forward)	CACGAACCACGGCACTGATT
TBP (reverse)	TTTTCTTGCTGCCAGTCTGGAC
TBP (probe)	VIC-TGTGCACAGGAGCCAAGAGTGAAGA-BHQ1
siRNAs	
siZNFx1#1	5′-GAUGGAGAGUUACCACCAAdTdT-3′ 3′-dTdTCUACCUCUCAAUGGUGGUU-5′
siZNFx1#2	5′-GGAAGGAGCAACAGUGAAAdTdT-3′ 3′-dTdTCCUUCCUCGUUGUCACUUU-5′
siZNFx1 #3	5′-GAGCAAAGUUAACAAAUCUdTdT-3' 3′-dTdTCUCGUUUCAAUUGUUUAGA-5'
siRHPN2#1	5′-CCGGAGUAAAUUGCAGAAUdTdT-3′ 3′-dTdTGGCCUCAUUUAACGUCUUA-5′
siRHPN2 #2	5′-GAAGGAAAGUAACCAAGAAdTdT-3′ 3′-dTdTCUUCCUUUCAUUGGUUCUU-5′
siRHPN2 #3	5′-UGGUGACAAUUAUGACUUUdTdT-3' 3′-dTdTACCACUGUUAAUACUGAAA-5'
siSTK11IP#1	5′-GGAUGGGAUUAGACAGUGAdTdT-3' 3′-dTdTCCUACCCUAAUCUGUCACU-5'
siUNC45A	5′-GAGAAGGUGCGAUACAUGUdTdT-3' 3′-dTdTCUCUUCCACGCUAUGUACA-5'

### RT and qPCR analysis

Total RNA was extracted from cells using phenol–chloroform method according to Chomczynski and Sacchi [19]. RNA was treated with DNAseI (Thermo Fisher Scientific, USA) and reverse transcribed using ImProm-II™ Reverse Transcription System (Promega, USA). The absence of DNA contamination was confirmed by qPCR with genomic loci primers. qPCR experiments for target genes were performed using EvaGreen® Dye (Biotium) and SmarTaq DNA Polymerase (Dialat) in StepOne instrument (Applied Biosystems). All PCR amplification reactions were run in triplicates for each cDNA sample. For normalization, we used expression levels of four reference genes (B2M, HPRT, TFRC, TBP) measured by multiplex qPCR. Primer sequences for reference genes and genes of interest are listed in Table 1.

### Cell growth assay

Cell viability was determined in 3-(4,5-Dimethyl-2-thiazolyl)-2,5-diphenyl-2H-tetrazolium bromide (MTT) assays. In brief, 10^3^ cells were transfected in 96-well plates in five replicated wells. After 24 h, cells were trypsinized, counted and seeded at 2×10^2^ cells/well. Every 24 h, five wells with cells were treated with MTT solution and incubated 3 h at 37 °C. After removal of media, formazan pellets were dissolved in 200 μl of DMSO. For each pellet, absorbance of formazan solution was analyzed at 570 nm in Multimode Plate Reader (PerkinElmer).

### Cell migration assay

Cell migration was assessed by wound-healing assay as described in [20]. In brief, cells were transfected in five replicates at 12×10^3^ cells/well in 96-well plates. 24 h post transfection cell mat was wounded by 200ul pipette tip and photographed every 2–5 h for up to 24 h in total. Photos were processed with software ImageJ [22]. The slope of linear fit curve was used to compare the rates of cell migration.

### Statistical analysis

All statistical analyses were performed using STATISTICA 8.0 software. Measurements were obtained at least in triplicates. Results are reported as the mean ± standard deviation. The Mann–Whitney U and the Kolmogorov–Smirnov tests were used to compare groups of samples. *p* < 0.05 was considered as statistically significant.

## Results

### Analysis of essential genes for melanoma surviving

We performed analysis of the 91 genes previously identified in silico as essential for survival of cutaneous melanoma [10]. FANTOM5 analysis of these showed that only 44 of them are expressed in melanoma cell lines. If a gene is essential for human cell surviving, it likely remains intact. All 44 genes were examined for their profiles of association with human diseases in the deCODE database. Only 18 of these 44 genes were free of any disease association. Next, we employed knowledge-based algorithms to identify known functions of these 18 selected genes and output of any previous knockdown experiments in any cell lines. Many of these remaining genes encoded the products associated with plasma membranes. Since membrane proteins may be involved in immune system evasion in vivo [21] and, therefore, cannot be adequately evaluated for their effects on surviving of melanoma cells in vitro, respective genes were discarded from further analysis. As a result, 10 most interesting and verifiable genes remained; for 7 of them no knockdown data were available for any melanoma cell model.

These 7 genes were analyzed for their expression levels in A375, G361, Sk-mel-1 and Sk-mel-5 melanoma cell lines by qPCR. Four genes that were expressed at detectable level at least in one of these cell lines were selected. Some of them were previously demonstrated to play a role in other cancers (UNC45A, STK11IP), and some were not yet characterized (RHPN2 and ZNFX1). These genes entered further experimental validation.

### Knockdown experiments

**Fig. 1:**
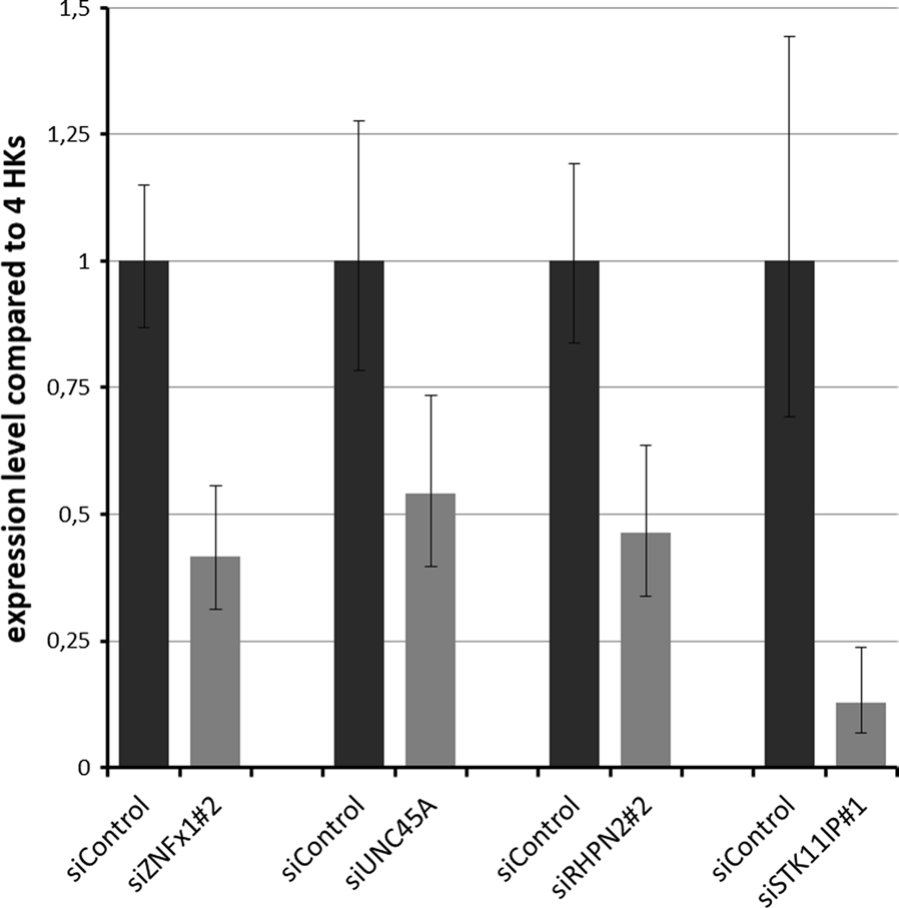
The levels as a knockdown of target genes by siRNAs were assessed by RT-qPCR. Samples treated with control siRNA are referred as baseline. Error bars reflect SD

For each of the genes, namely ZNFx1, RHPN2, STK11IP and UNC45A, from 1 to 3 siRNAs were designed and tested in melanoma cell line A375 (Fig. 1). For designing, we employed home script which parses every 20 nt of the mRNA sequence of the gene by tiling and apply to them the rules listed in [17]. Post-knockdown levels of cognate mRNAs were quantified using RT-qPCR in the target cells and in the cells transfected with scrambled siRNA control. For each of the gene, the most efficient siRNA was then used to examine the functional effects of the knockdown in viability, migration and proliferation assays.

**Fig. 2:**
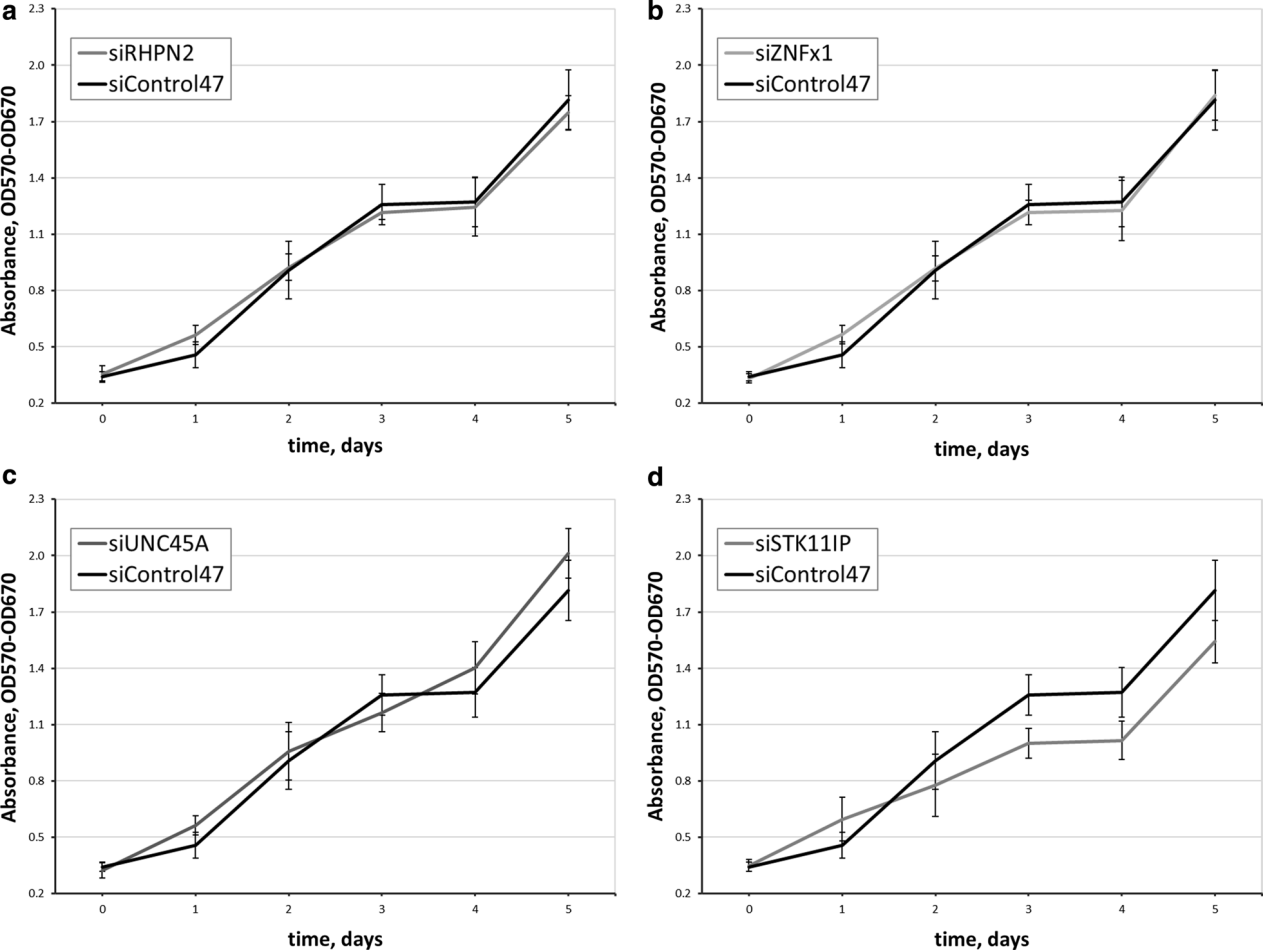
Effect of KD of the genes on cell viability evaluated by MTT assay. Five repeats of each experiments sample were conducted. Error bars represent SD . **a**–**d** Cell viability after RHPN2, ZNFx1, UNC45A or STK11IP knockdown, respectively

For viability assays, one thousand cells per well in 96-well plate were transfected and cultivated in standard conditions for 6 days. Every other day we assessed the viability by an MTT test. Proliferation assays were performed every 12 h during 45–60 h range by flow cytometry. Prior to cytometry, cells were tripsinized and resuspended in PBS. Proliferation assay showed no significant differences in cell counts between knocked down and control cells (data not shown). Viability assay also revealed no statistically significant changes in cell survival (Fig. 2).

**Fig. 3:**
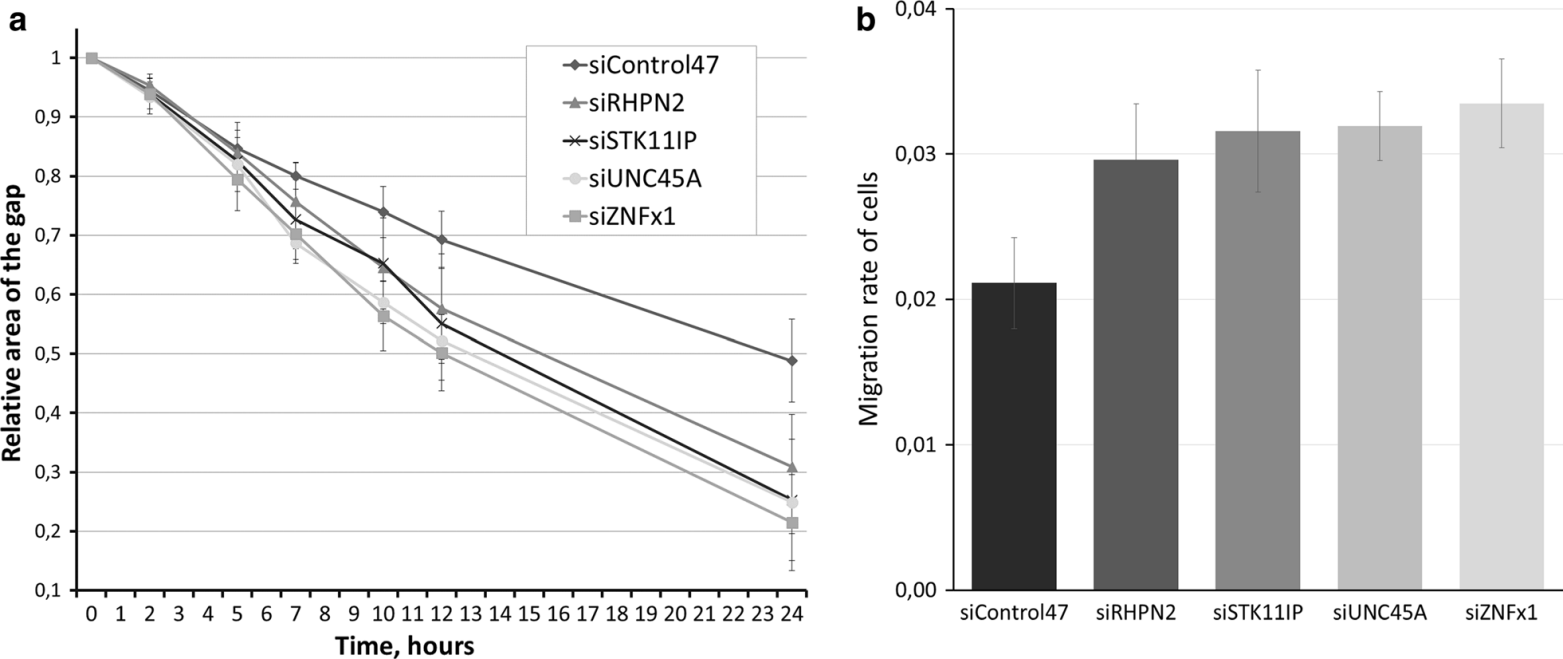
Results of wound-healing assay. **a** Percent of remaining gap between cell carpet front. Area of gap at first time point is considered 1. **b** Speed of sell migrating calculated by the slope of A graphics. Error bars represent SD

Then, we analyzed the migrating rate in cells transfected with target and control siRNAs using a wound-healing assay. Our results show that knockdown of any of the target genes, ZNFx1, RHPN2, STK11IP and UNC45A, accelerates the speed of migration of cells (*p* < 0.05) (Fig. 3).

## Discussion

Recent advantages in DNA sequencing facilitate analyzing genomes of human tumors. Instead of considering each tumor as a result of a series of point mutations in oncogenes or tumor suppressor genes researches regard the malignancies as completely different tissue type [22]. Cancer cells pass through rigorous natural selection accompanied by genome reorganization [23, 24]. In any given cancer cell, a majority of mutations are the passengers, or innocent bystanders rather than drivers. T They do not improve ability of the tumors to survive, and, therefore, are a subject of either a negative selection or a selective sweep [25]. Nevertheless, a variety of true driver mutations were discovered. The most common way of searching for driver mutations is to compare genomes of cancer samples and adjusting healthy tissues following by identification of genes repeatedly mutated in many samples of human tumors cancer [9]. This approach may be complemented by an approach which is exactly opposite of one described above, namely, by looking for genes which never get any mutation in the tumors of a given type, with an assumptions that these mutations would render respective cells non-viable or non-competitive [11]. Execution of this approach in the model of human cutaneous melanoma revealed 91 genes which could be essential for melanoma surviving. Therefore, knockdown experiments in melanoma cell lines were performed to evaluate this theory.

According to Fantom5 dataset, just 44 of these genes) were, in fact, expressed in melanoma cell lines. This discrepancy may be explained by the differences could be explained by a difference between expression profiles of real tumors collected from patient and the immortalized cell lines explored in Fantom5. Further whittling down the gene list was performed by exclusion the genes previously associated with any human disease. No consensus trends were observed in subsequent analysis of 18 remaining genes, with some of them behaving in accordance with initial hypothesis, and some in exactly opposite fashion. For instance, previously described knockdowns of UNC45A in ovarian cancer and in myoprogenitor cells led to a decrease in their ability to proliferate [26]. Similarly, shRNA inhibition of PTK2B resulted in the reduction of the growth of multiple myeloma tumors in vivo and a decrease in cell proliferation, cell-cycle progression, and adhesion ability in vitro [27]. On the other hand, some genes behaved as cancer drivers. For example, downregulation of MYCT1 was observed in a majority of studied gastric carcinoma samples, in accordance of its ability to promote apoptosis of gastric carcinoma cell lines when overexpressed [28]. In a similar fashion, overexpression of TGM5 was reported as inducing cell death [29].

We have taken onto account that for some genes effects on cell survival may be tissue-specific, and some other genes may play a role in survival of the cells only in the contexts of multicellular 3D structures, or when cancer cell would interact with immune cells. To minimize effects of the interaction taking place at the cell-to-cell interfaces, we have reduced the list to seven genes, by excluding all genes encoding for the proteins expressed on a surface of human cells.

After analyzing expression levels for these 7 genes in available melanoma cell lines sk-mel-1, sk-mel-5, G361 and A375, four genes with the highest expression levels all over, and in the A375 cell line in particular, were selected for knockdowns. Notably, for all four selected genes, namely, UNC45A, STK11IP, RHPN2 and ZNFX1, detected expression levels were extremely low. Nevertheless, we assumed that these genes may be important, for example, they may serve as transcription factors, or other regulatory molecules, and proceeded with wet-lab experiments.

The results on knockdown experiments show that the selected genes are not essential for melanoma cells viability or proliferation, but evidently exert their influence at the speed of the cell migration, and, possibly, other processes, not yet explored experimentally. None of these genes were extensively studied by other research groups, hence, current study does add to the current body of evidence for their roles in the functioning of the human body.

Gene UNC45A encodes co-chaperone of heat shock protein 90 (Hsp90). This gene is essential for certain types of cancers cells, including breast carcinoma, but is dispensable for normal cells [30], and for some other cancer types. Its connection to cell motility phenotypes are numerous as its function as a mitotic spindle-associated protein that destabilizes microtubules (MT) activity [31]. In ovarian tumor cells, its depletion restores sensitivity to pactitaxel [31]. Notably, paclitaxel is one of the prominent suppressors of chronic inflammation and supporters of immunosurveillance, and is currently in trials as anti-melanoma adjuvant. It is tempting to speculate that the maintenance of UNC45A is essential for masking of highly immunogenic melanoma cells from the restrictive actions of immune system.

Gene STK11IP encodes a leucine-rich repeat containing cytoplasmatic protein functionally linked to the Peutz-Jeghers syndrome kinase LKB1, and capable of interacting with the TGFbeta-regulated transcription factor SMAD4, and a formation of a ternary complex of LKB1- STK11IP-SMAD4 [32]. LKB1 is a bioenergetic sensor that controls cell metabolism and growth by phosphorylating and activating AMP-activated Kinase (AMPK) in starving cells, but also is a key player in regulation of the immune system, which dampens proinflammatory responses in macrophages and maintenance of immunosurveillance [33]. Accordingly, LKB1 deficient mice are less capable of controlling melanoma tumor growth due do an inherent defect in their DC-driven immunity and tolerance [33]. If the effects of a product of STK11IP antagonize that of LKB1, than the lack of STK11IP mutations in melanoma cells may be expected. On the other hand, in melanoma cells, loss of LKB1 promotes cell invasion and migration through upregulation of MMP-2 [34]. Loss of another component of LKB1-STK11IP-SMAD4 complex may lead to enhancement of the migration cells, if the effects of this component are synergistic with that of LKB1. While the function of STK11IP at the level of the whole body may not be relevant to the melanoma cell model in vitro, the observed pro-migratory effects of STK11IP knockdown align with a tumor suppressor and anti-metastatic function of its major protein partner, LKB1.

Rhophilin Rho GTPase binding protein 2 (RHPN2) drives mesenchymal transformation of malignant gliomas [35] and is likely to participate in actin skeleton organization [36]. With that, its involvement in preventing an increase in the motility of the cells and in their migration is not surprising. More intriguing, similarly to gliomas, melanomas rely on phenotype switching between differentiated/proliferative and stem-cell/invasive transformation states as a key to their intra-tumor heterogeneity and resistance to treatment [37]. While the promotion of the migration observed in the RHPN2 deficient cells was certainly not expected as it directly contradicts the data obtained in neuroprogenitor cells [35], protecting the central EMT driver from unwanted mutational events may certainly be expected.

Zinc finger NFX1-type containing 1 (ZNFX1) encodes an interferon (IFN)-stimulated, mitochondrial-localized dsRNA sensor capable of restricting the replication of RNA viruses [38]. While its antiviral functions may not be relevant to the progression of human tumors, its antisense lncRNA ZFAS1 (zinc finger antisense 1) is well known for promoting growth of melanoma and other tumors [39]. Specifically, in melanoma, knockdown of ZFAS1 was shown to reduces migration, invasion, and the markers of epithelial-mesenchymal transition [39]. It is very likely that the knockdown of ZNFX1 resulted in the proportional increase in the levels of its antisense, thus, explaining the respective increase in melanoma cell migration.

## Conclusion

In sum, our finding fail to confirm the hypothesis of Pyatnitskiy et al. Although some genes in certain distinct tumor types are hypomutated, they remain dispensable for the survival of these tumor cells. In accordance with our findings, Pyatnitskiy et al. have corrected their predictive model and published a novel in silico gene set [40].
